# The recovery trajectory of adolescent social defeat stress-induced behavioral, ^1^H-MRS metabolites and myelin changes in Balb/c mice

**DOI:** 10.1038/srep27906

**Published:** 2016-06-10

**Authors:** Handi Zhang, Gen Yan, Haiyun Xu, Zeman Fang, Jinling Zhang, Jie Zhang, Renhua Wu, Jiming Kong, Qingjun Huang

**Affiliations:** 1 Mental Health Center Shantou University, Shantou, China; 2 Affiliated Hospital, Jiangnan University, Wuxi, China; 3 The 2^nd^ affiliated Hospital, Shantou University, Shantou, China; 4 Department of Human Anatomy and Cell Science, University of Manitoba, Winnipeg, Canada

## Abstract

Adolescent exposure to social stress precipitates emotion-related disorders and affects the development and function of medial prefrontal cortex (mPFC). However, this adversity-induced behavioral and neurological changes remain not fully explored. Adolescent Balb/c mice were subjected to intermittent social defeat stress during postnatal days 28 to 42. Proton magnetic resonance spectroscopy (^1^H-MRS) measurements, behavioral tests and immunohistochemistry were performed one day or 3 weeks after the last stress episode. Defeated mice exhibited hypoactivity and social avoidance with the latter lasting into the early adulthood, while the anxiety level was unchanged. Social defeat experience lead to temporary decreases in the levels of total creatines (Cr + pCr) and Glx (Glu + Gln), but a delayed increase of N- acetylaspartate (NAA) levels. These alternations were accompanied with a persistent reduction of myelin basic protein expression although the number of mature oligodendrocyte did not change. These findings provide evidence that adolescent adverse social experience permanently impairs the emotion-related behavioral performance and induces biochemical and molecular changes in the brain which at least lasts into early adulthood, thus enhancing our understanding of the neurobiology of social defeat stress. Our finding also implicates that NAA signals on MRS may reflect myelin status.

Childhood and adolescent adversities are associated with the increased risk of developing later psychopathology^1^,^2^,^3^, including major depressive disorder^4^, anxiety disorders, impaired social behavior^5^,^6^ and cognitive functions^6^,^7^. However, the underlying neurobiological mechanism remains largely unknown.

The medial prefrontal cortex (mPFC) is a late-maturing forebrain structure involved in higher cognitive processes and emotional regulation. Substantial evidence points to the mPFC as a key brain region, along with the amygdala and hippocampus affected in stress-related disorders such as anxiety and major depressive disorders^8^,^9^. As the mPFC exhibits a prolonged postnatal developmental trajectory, it is especially vulnerable to early-life stress^10^.

Magnetic resonance spectroscopy (MRS) is a non-invasive method to measure levels of cerebral metabolites *in vivo* and has been widely applied in research and clinical practice with psychiatric patients^11^. Metabolite changes in prefrontal cortex measured by MRS have been reported in stress-related disorders including depression^12^, anxiety disorders^13^,^14^, obsessive-compulsive disorder^15^, post-traumatic stress disorder (PTSD)^16^ and bipolar disorder^17^. The MRS findings suggest that prefrontal cortex is one of the targets susceptible to childhood and adolescent stress^18^,^19^. However, to our knowledge, only one clinical study has investigated the long-term effect of childhood and adolescent stress on later levels of brain metabolites, and found a significant decrease of the N-acetylaspartate (NAA) to creatine (Cr) ratio in anterior cingulate cortex (ACC)^20^. While the results of clinical studies have been widely appreciated, the limitations of them are obvious, including cross-sectional data, no control for genetic factors, and the comorbidity of psychiatric disorders. These prevent us from claiming a causal relationship between stress and neurochemical changes. To overcome these limitations, animal studies are necessary in detecting the direct impact of early adversities on the development and function of brain^21^. Recent studies found that adult animals with experience of early-life stress displayed lower levels of NAA in ACC^22^ and hippocampus^23^ compared with controls. But, it is unknown if adolescence stress exerts similar effects on neurochemistry indices of the brain.

Adolescence is the period during which the brain is still undergoing maturation process exemplified by myelination process in the prefrontal cortex during this period^24^. It has been found that early adolescence is a critical period for oligodendrocyte/myelin maturation in mPFC in rodent, and social isolation during this period significantly impaired oligodendrocyte maturation and myelination in this brain region^25^. In this study we examined effects of social defeat stress during early adolescent period on the behavioral performance of mice and on their brain metabolites, and myelination in mPFC.

## Results

### The intermittent social defeat stress-induced hypoactivity, but not social avoidance, recovered in three weeks after the stress

**Figure 1:**
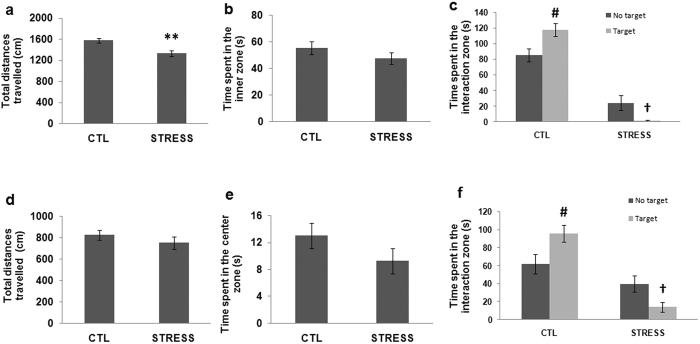
The immediate and long-lasting behavioral effects induced by intermittent social defeat stress during adolescence in Balb/c mice. When evaluated right after the last session of stress, mice in the stress group travelled less distances (**a**), but spent comparable time in the centre zone in the open field test (**b**) compared to mice in the control group. The defeated mice showed intense aversion to a social target as indicated by the less time spent in the interaction zone compared to no target presented, while control mice behave oppositely (**c**). When evaluated 3 w later, the total distances travelled (**d**) and the time spent in the center zone (**e**) in the open field test are comparable between the two groups of mice, whereas the previously defeated mice still showed social avoidance behavior and the control mice showed increased social interaction (**f**). Data are expressed as mean ± sem (n = 17 ~ 19 per group), **p < 0.01, STRESS vs. CTL, ^#^p < 0.05, target vs. no target in CTL, ^†^ <0.05, target vs. no target in STRESS.

Emotion-related behaviors including locomotion activity, anxiety-like behavior and social avoidance were analyzed shortly after the last stress episode, and the recovery of these behaviors were evaluated three weeks later. Repeated measures two-way ANOVA indicated that a significant interaction of stress and time on total distance travelled in open-field test (F _1, 34_ = 5.68; P < 0.05). Post-hoc comparison showed a significant difference between control and stress group at one day after the last stress session (Fig. 1a), but not three weeks later (Fig. 1d). For time spent in the inner zone in open-field test, the statistical analysis showed no significant interaction of stress and time (F _1, 34_ = 0.23; P > 0.05), and post-hoc comparison found no significant difference between control and stress group at either time points (Fig. 1b,e).

Shortly after the last stress episode, In the social avoidance test, control mice spent significantly more time in the interaction zone with an unfamiliar mouse (target) present than without any mouse (no target) (Fig. 1c); in contrast, the mice in the stress group spent significantly less time in the interaction zone when a social target was present (target) than no social target there (no target) (Fig. 1c). Social avoidance test was retested three weeks later in the early adulthood of mice. Mice in stress group still spent markedly less time in the interaction zone when an unfamiliar mouse was present (Fig. 1f) compared to no target, whereas mice in the control group spent significantly more time in the interaction zone when a social target was present than no social target was there (Fig. 1f).

### The intermittent social defeat stress induced delayed increase of NAA and temporary decrease of total creatines (Cr + pCr) and Glx (Glu + Gln) which recovered in 3 weeks after stress

**Figure 2:**
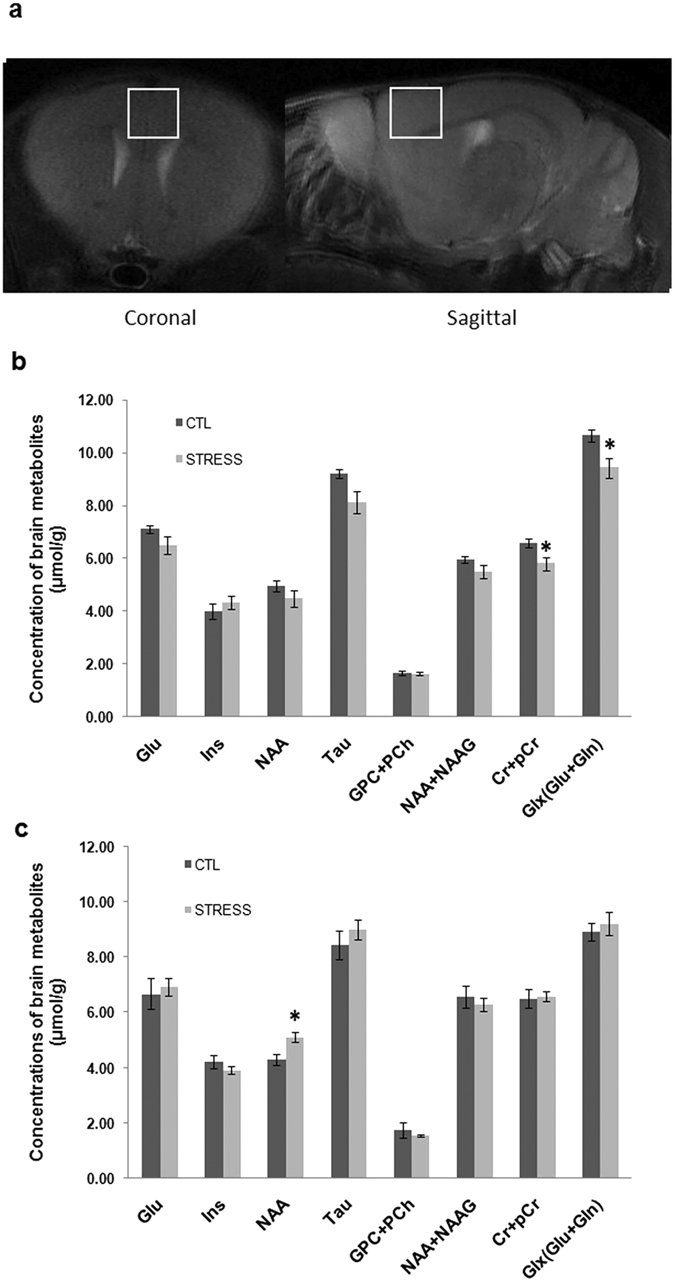
The immediate and delayed effects of intermittent social defeat stress during adolescence on brain metabolites in medial prefrontal cortex (mPFC). (**a**) Location of the volume of interest (VOI) in a coronal and sagittal section in a brain of Balb/c mouse. (**b**) The brain metabolite concentrations measured by MRS in the control and defeated mice right after the end of stress. (**c**) The brain metabolites measured in the two groups 3 w later. Data are expressed as mean ± sem (n = 8 ~ 10 per group), *p < 0.05, STRESS vs. CTL.

Levels of brain metabolites were measured by MRS shortly after the end of stress and 3 weeks later. Of the analyzed neurochemicals and neurochemical combinations in mPFC, statistical analysis indicated that a significant interaction between stress and time on NAA levels (F _1, 17_ = 7.91; P < 0.05), Glx levels (F _1, 17_ = 5.52; P < 0.05) and Taurine levels (F _1, 17_ = 4.78; P < 0.05). Post-hoc comparisons revealed that the concentrations of total creatines and Glx showed a significant decrease right after the last session of stress (Fig. 2b). But, these changes returned to the levels of control group in the second MRS which was done three week after the last stress episode (Fig. 2c). A reduced trend was found on Taurine levels while it did not reach the statistical significant level. NAA was not changed shortly after the last stress episode (Fig. 2b), but increased at 3 weeks after the end of stress (Fig. 2c). No significant changes were found for other neurochemicals and neurochemical combinations between the two groups (Fig. 2b,c).

### The incomplete recovery of the intermittent social defeat stress-induced decrease in myelin basic protein (MBP)-immunoreactivity in mPFC

**Figure 3:**
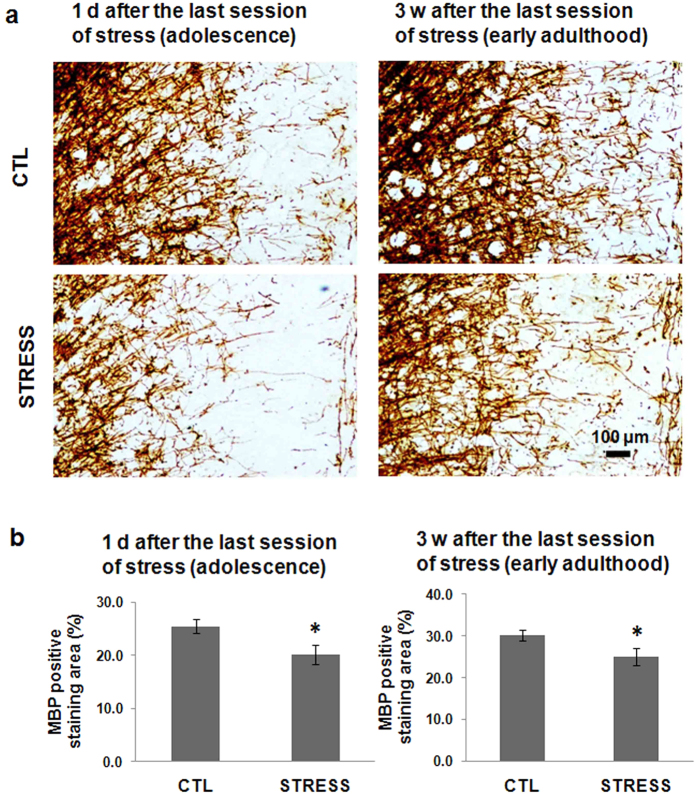
The short- and long-term effect of intermittent social defeat effect during adolescence on myelin basic protein (MBP) immunohistochemical staining. (**a**) Representative images of MBP staining in the mPFC. (**b**) The bar graph shows quantitative data of the percentage of MBP-positive immunostaining area over the examined region. Dara are expressed as mean ± sem (n = 4 ~ 5 per group), *p < 0.05, STRESS vs. CTL.

MBP, a structural protein in myelin sheath, was labelled to detect the status of myelin. As shown in Fig. 3a, intermittent social defeat stress exposure during early adolescence resulted in a significant decrease of MBP-positive staining in mPFC immediately after the last session of stress. This reduction of MBP expression was still obvious when evaluated 3 weeks later in the early adulthood of mice (Fig. 3a). Statistical analysis showed a significant difference in the percentage of positive MBP staining area in a segment of mPFC between the normal control group and the stress group (Fig. 3b).

**Figure 4:**
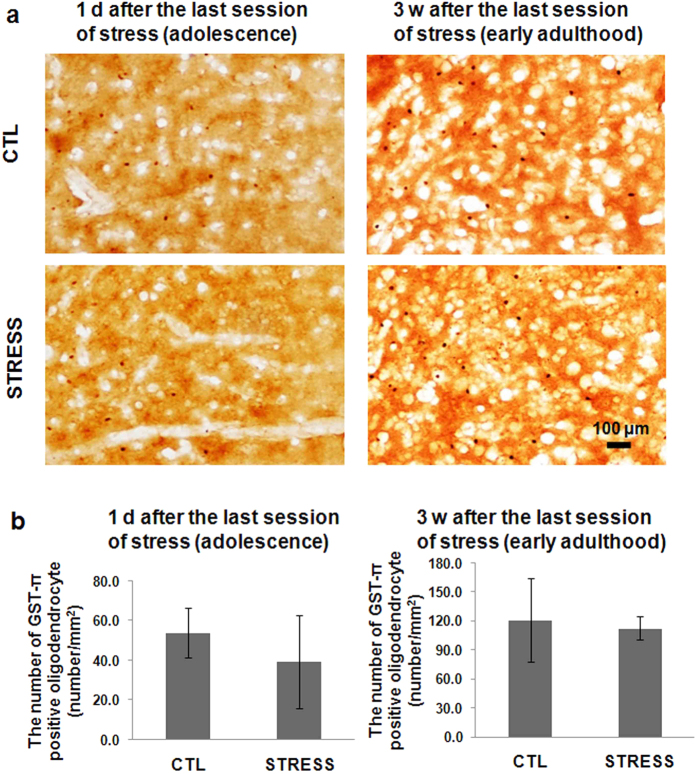
The short- and long-term effect of intermittent social defeat effect during adolescence on the number of mature oligodededrocyte. (**a**) The representative images show the π form of glutathione-S-transferase (GST-π) immunostaining in the mPFC. (**b**) The bar chart shows the quantitative data of GST-π positive cells. Dara are expressed as mean ± sem (n = 4 ~ 5 per group).

The π form of glutathione-S-transferase (GST-π), an oligodendrocyte-associated enzyme, was used to identify mature oligodendrocyte in mice. At either time point, intermittent social defeat stress during early adolescence did not cause evident oligodendrocyte loss in the mPFC, as the control and stress group showed similar numbers of GST-π positive cells (Fig. 4a). There was no statistically significant difference between the stress and the control group (Fig. 4b).

## Discussions

In this study, we used murine social defeat stress model to investigate the consequences of stressful experience during adolescence on emotion-related behaviors, brain metabolites and oligodendrocyte/myelination pathology. We found: 1) adolescent social adverse experience changed levels of cerebral metabolites in mPFC; 2) adolescent social adverse experience altered some of emotion-related behaviors in mice; 3) adolescent social adverse experience significantly decrease the expression of MBP in mPFC, but had no effect on the number of mature oligodendrocyte.

Intermittent exposure to social defeat stress during early adolescence caused a lasting social avoidance behavior in defeated animals. Meanwhile, these animals also showed a transient reduction in locomotor activity, but no changes on anxiety status. A reduction of locomotor activity may represent a decline of non-specific excitability level of stressed mice^26^. Another possibility is that defeated animals displayed hypoactivity because of the loss of interest to the novelty of open field, which may be related to hedonic deficit, a core symptom of depression^27^. Previous studies showed that decreased locomotor activity coincide with depressive-like behaviours in stressed animals^28^,^29^,^30^. To clarify this issue, further studies should investigate the depressive-like behaviors in this model. Previous studies reported contradictory effects of chronic social defeat stress on locomotion in adolescent animals^29^,^30^,^31^,^32^,^33^,^34^. Similarly, its effect on anxiety status in defeated adolescent animals is also mixed^29^,^30^,^31^,^32^,^33^,^34^,^35^. Contrast to our result, adolescent animals exposed to 10-day continuous stress or longer displayed increased anxiety-like behaviour in open field test^29^,^30^ or elevated-plus maze test^32^,^33^ shortly after the end of stress. Studies focusing on the long-term effect found no change in anxiety levels^33^,^35^ or increased anxiety^31^. One study examining the immediate effect of social defeat stress found 5-min daily stress did not induce alteration in anxiety status, whereas 10-min daily stress significantly increased anxiety level, which indicated that the effect on anxiety status depend on stress intensity^34^. The discrepancy on the locomotion and anxiety levels among these studies may also relate to the differences in stress regimen, timing, housing conditions and the setting-up of the test^26^,^36^.

The most prominent behavioral change in this study is social avoidance exhibited by defeated mice, and this change did not recover even 3 weeks after the last session of stress. This finding is consistent with that of previous studies showing enduring social avoidance displayed by defeated animals to an unfamiliar animal whose physical size was similar to dominant aggressors^29^,^30^,^32^,^37^,^38^. Meanwhile, in our study animals’ anxiety status were not altered in open field test, indicating that social avoidance is not a consequence of altered general anxiety status. Social avoidance behavior may reflect a conditioned fear response to social stimuli or a lack of social motivation^39^,^40^. Previous studies in adolescent animals showed that social defeat stress lead to persistent fear responses like freezing and increased risk assessment behaviors^31^ or depressive-like behaviors^29^,^33^, indicating that they are possible reasons why animals displayed social avoidance behavior. Further studies should explore the underlying neurobiological mechanisms for the long-persistent social avoidance induced by adolescent social adverse experience.

The MRS results demonstrated that, accompanying with behavioral changes, social stress experience during early adolescence produces immediate and delayed changes in levels of cerebral metabolites in mPFC. We observed an immediate decrease in levels of total creatines and Glx, and they completely recovered to normal levels three weeks later. Total creatines are the marker of cellular energy metabolism, reflecting the general healthy status and integrity of neural cells^11^. Glx is involved in energy metabolism and neural activity^11^. The parallel decreases of these metabolites in mPFC perhaps reflect compromised mPFC neural integrity in this model. Consistent with this inference, animals exposed to stress during adolescence display altered neural activity and synapse plasticity^41^, dendritic atrophy and reduction of dendritic spines^42^. Consistently, our immunohistochemistry staining results of MBP reduction indicates a possible damage of myelin integrity in this brain region.

Interestingly, NAA level was not altered right after the end of stress, but significantly increased three weeks later when measured in the early adulthood. Although NAA is mainly synthesized in neuron and considered as a marker of neuronal integrity^11^, its degradation is majorly carried out by oligodendrocyte-located aspartoacylase (ASPA)^43^. The deficiency of ASPA is the cause of a recessive inherited disease, Canavan’s disease, which is characterized by NAA accumulation and myelin defect^44^. These studies indicate that oligodendrocyte plays an important role in NAA metabolism, and change of NAA levels may also reflect dysfunction of oligodendrocyte/myelin. Given that early adolescence is a key period for oligodendrocyte/myelin maturation in mPFC in rodents and the process is vulnerable to stressful experience^45^, we speculate that the increased NAA levels observed in the second MRS measure may reflect an abnormity of oligodendrocyte/myelin maturation in this brain region. In support of this view, NAA increment is considered to reflect myelin deficit in Canavan’s disease^46^,^47^. This speculation is also supported by the immunostaining results showing that MBP expression significantly reduced in mPFC in defeated animals. But, why NAA levels were not changed right after the last session of stress when MBP immunoreactivity was already decreased? One explanation is that together with oligodendrocyte dysfunction right after the end of stress, there must be also a transient neuronal dysfunction as indicated by the disturbances of other metabolites. The neuronal and oligodendrocyte dysfunctions have opposite effects on levels of NAA, i. e. the former increases, but the latter decreases levels of NAA as reviewed earlier in this paragraph. Nonetheless, further studies are required to clarify the underlying neurobiological mechanism for the metabolite changes measured by MRS.

The MBP immunoreactivity in mPFC was decreased by adolescent exposure to adverse social experience, but no oligodendrocyte loss, indicating that the myelination process was impeded in this brain region. This result is consistent with a previous study showing that social isolation during early adolescence did not change the number of mature oligodendrocyte but reduced the expression of MBP^25^. Further study should be conducted to see whether the morphology of mature oligodendrocyte is altered in our stress model as this is the case in social isolation model^25^. A previous human study found pubertal verbal stress is associated with adult decreased fractional anisotropy value in several brain regions, suggesting that oligodendrocyte and myelination are possible targets of the early-life stress^48^. Social isolation in early adolescent period lead to deficit in myelination in murine mPFC, and ErbB3 signalling pathway may mediate this effect^25^. Together with these previous studies, our study demonstrated that, social defeat stress, a different stress model from social isolation, also causes the deficit of myelination development in mPFC when administrated during early adolescence. As social defeat is a different stress paradigm from social isolation, it may exert its effect on myelination via different neurobiological mechanisms. Further study is needed to investigate the underlying mechanism for the myelination impairment in social defeat stress model.

In our model mPFC myelin pathology was concurrent with social avoidance displayed by defeated mice, which indicates that myelin integrity may be necessary for the normal social behavior in both adolescents and adults. Consistent with this view, the maturation of oligodendrocyte in mPFC during adolescence has been demonstrated necessary for normal social interaction in adult mice^25^,^49^. However, other studies found neural activity alternations in mesocorticolimbic pathway, including mPFC, nucleus accumbens (NAc) and ventral tegmental area (VTA), was linked to social avoidance induced by social defeat stress in adults^50^,^51^,^52^. Furthermore, the mPFC-NAc projection regulated social avoidance^50^. In this context, one scenario is that impaired myelin changes conduction of mPFC axons thus disturbs the reciprocal innervations between mPFC and NAc and leads to social avoidance behavior. Since impaired oligodendrocyte function also changes dopamine neurotransmission^53^, another possibility is that myelin defects affect dopaminergic function thus contributes to aberrant social interaction^54^.

In summary, social defeat stress during early adolescence led to enduring social avoidance behavior, a common symptom in stress-related disorders, like PTSD, depression and social anxiety, suggesting that adolescent social stress is a direct environmental risk factor in pathogenesis of these disorders. Furthermore, adolescent social stress caused both a temporary and a delayed neurochemical changes in mPFC, which are concurrent with an abnormal development in myelination in this brain region. These results suggest that social adversity experience during adolescence exerts immediate and long-lasting influences on behaviors, neurochemistry and myelination, which are relevant to the symptoms and pathophysiology of stress-related psychiatric disorders.

## Methods

### Animals

Adolescent male Balb/c (aged 3 weeks) mice were purchased from Vital River Laboratories (Beijing, China). They were housed in groups (3–5 per cage) with a regular light-dark cycle at controlled temperature (22 °C ± 1 °C) and were given standard diet and water ad libitum. CD1 retired breeders (age of 8–10 months) purchased from Vital River Laboratories (Beijing, China) were used as resident aggressor and were individually housed. Prior to the experiment, CD1 mice were screened for their levels of aggression and mice that attacked against a partner within 30 seconds were used for the intruder-resident social defeat procedure. The CD1 mice showing little aggressiveness were used as social target in social avoidance test. All animal procedures were in accordance with the guideline of the Animal Care and Use Committee of Shantou University Medical College and approved by the Animal Ethics Committee (SUMC2013-031).

### Experimental procedures

**Figure 5:**
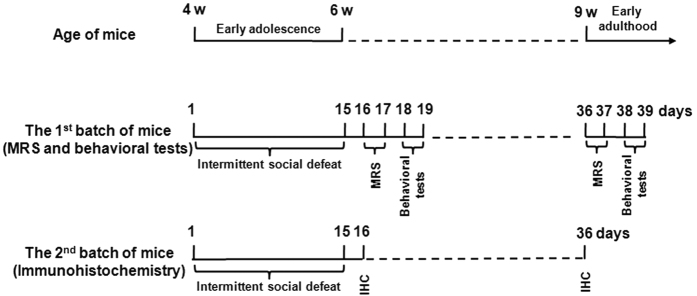
Experimental procedures. The top diagram shows the ages of Balb/c mice used in the experiments. The middle diagram displays the experimental process for the first batch of Balb/c mice which were firstly exposed to intermittent social defeat stress during the early adolescence, followed by magnetic resonance spectra (MRS) measurements and behavioral tests immediately and 3 weeks later after the last session of stress. The bottom diagram displays the experimental process for the second batch of Balb/c mice which were sacrificed for immunohistochemical staining 1 d and 3 w after the end of stress, respectively.

Two batches of Balb/c mice were used: the first batch was used for MRS measurement and behavioral tests, and the second batch was for immunohistochemistry. The experimental procedures are shown in Fig. 5. Briefly, after acclimatization for 7 days, Balb/c mice were randomly assigned to either a control or stress group (In the first batch: n = 17 ~ 19 per group; in the second batch: n = 10 per group). Mice in the stress group were subjected to social defeat stress once a day for consecutive three days followed by a day off. Over a course of 15 days, each one of mice in the stress group experienced 12 defeat stress episodes. The mice in the control group were handled at the same time without exposure to a resident aggressor. For the first batch of animals, 10 animals in each group were subjected to MRS measurement one day after the last stress episode, and then one day after MRS measurement all the mice were subjected to behavioral tests, including open-field test and social avoidance test in order. There was an interval of 24 h between the two behavioral tests. After the last behavioral test, all the mice were reared (3 ~ 5 per cage) under the regular housing conditions without any stress exposure for another 16 days. Then, they were subjected to the second procedures of MRS measurement and behavioral tests. For the second batch of animals, one day after the last stress episode, 5 mice in each group were sacrificed under deep anaesthesia and their brains were processed for immunohistochemical staining to detect oligodendrocyte/myelin pathology; whereas the rests (5 mice in each group) were reared under normal conditions for another 20 days until sacrifice and processed for the same immunohistochemical staining.

We choose to perform MRS scan before behavioral tests in order to exclude the potential confounding effects of behavioral tests on MRS measures. The potential effects of MRS manipulation on performances of animals in the behavioral tests were also examined by comparing the behavioral performances of those subjected to MRS (n = 10) and the remained others (n = 7) received no MRS manipulations. No significant differences were found between the MRS manipulation and non-MRS manipulation mice in either control and stress groups. Indeed, no evidence, to our knowledge, has showed that MRS procedure alters the performance of animals in the behavioral tests.

### Social defeat stress

Social defeat stress was induced by using intruder-resident procedure as reported in a previous study^52^ with minor modifications. Briefly, each Balb/c mouse was introduced into the home cage of an unfamiliar resident CD1 aggressor for 1 min and was physically defeated. After 1 min of encounter, the intruder and resident were maintained for sensory contact for 30 min in two parts separated by a wire mesh barrier in a cage. After each social stress episode, the Balb/c intruder was returned to their home cage. On the subsequent stress episodes, CD1 resident was changed every time to ensure the resident and intruder had never encountered on consecutive days.

### Magnetic resonance imaging (MRI) and proton MRS acquisition

*In vivo* MRI and spectra were acquired on a 7.0 T horizontal DriveDrive 2 MR system (Agilent Technologies, USA) with a 160-mm bore and 400 mT/m actively shielded gradient coil. A dedicated animal brain surface coil (Varian Medical Systems, Inc., Palo Alto, CA) was used as the radio frequency transmitter and the signal receiver. Mouse was anaesthetized by intraperitoneal injection of 2% pentobarbital in 0.9% NaCl at a dose of 80 mg/kg. The scan time for one animal was within 60 min.

A scout imaging was first acquired to verify the image quality and the subject position. T2-weighted image was obtained by using Rapid Acquisition with a Relaxation Enhancement (RARE) sequence with repetition time/echo time (TR/TE) = 5000/48 ms, slice thickness = 1.0 mm, matrix = 192 × 192). Then, the volume of interest (VOI) of 2.0 × 2.2 × 2.0 mm was positioned in mPFC (Fig. 2a). Localized shimming was performed automatically for the VOI to achieve a water spectrum width of 15–20 Hz. An ultrashort echo time stimulated echo acquisition pulse sequence was used for the acquisition of proton spectra in the VOI (TR/mixing time (TM)/TE = 5000/12.72/2.35 ms; the spectral width = 5000 Hz; number of excitations (NEX) = 320; scan time = 26 min). Water suppression was performed with variable pulse power and optimized relaxation delays. To compensate for eddy currents, we acquired a water reference scan, which also served as an internal reference for absolute quantification.

### Processing of ^1^H-MRS spectrum

*In vivo*^1^H-MRS data were analyzed using the Linear-Combination Model (LCModel) (Provencher 2001). Raw data were used as standard data input. The water-suppressed time domain data were analyzed between 0.2 and 4.0 ppm without further T1 and T2 corrections. The following seventeen metabolites were evaluated in the basis set: alanine (Ala), aspartate (Asp), creatine (Cr), γ-aminobutyric acid (GABA), glucose (Glc), glutamate (Glu), glutamine (Gln), glutathione (GSH), glycerophosphorylcholine (GPC), lactate (Lac), myo-inositol (mI), N-acetyl-L-aspartate (NAA), N-acetylaspartylglutamate (NAAG), phosphorylcholine (PCh), phosphocreatine (PCr), scyllo-inositol (Scy) and taurine (Tau). Their signal intensities were processed with water scaling for metabolite quantification. Besides individual metabolites, the sums of some metabolites (NAA + NAAG, Glu + Gln, GPC + PCh and Cr + PCr) were also included. The reliability of metabolite quantification was evaluated by standard deviation of the fits, which was expressed as a standard deviation (%) of the estimated concentration by Cramer-Rao lower bounds in LC Model. Our criterion for the reliability of the spectral fit was <20%. Only the measurements of the metabolites and their combinations that meet this criterion were included in the final statistical analysis.

### Open field test

Open field test was performed to assess locomotor activity and anxiety level of a mouse. Each mouse was initially placed at one corner of the open field (25 cm × 25 cm × 30 cm) facing the wall and allowed to move freely during the 5-min session. The travel paths of the animal were recorded by a video camera placed above the arena. The total distance travelled and the time spent in the central zone were analyzed by the video tracking system (DigBehav System, Yishu Co Ltd, Shanghai, China). The open field was cleaned with 70% alcohol after each trial.

### Social avoidance test

The test procedure was accordance to the previous report with a moderate modification^52^. This test was performed 24 h after the open-field test to measure approach and avoidance behavior toward an unfamiliar mouse (social target mouse). Social target mice were novel non-aggressive male CD1 mice as determined by pre-screening testing. The test was carried out in the same open-field arena used for open-field test (25 cm × 25 cm × 30 cm). A wire-mesh cage (6 cm × 8 cm × 8 cm) was positioned at one of the corners. Each mouse was introduced into the open field and its trajectory was video-tracked for two 2.5-min sessions which were separated by a 1-min interval. In the first session (“no target”) the open field contained an empty wire-mesh cage. In the second session (“target”), the conditions were identical except that a social target had been placed into the wire-mesh cage. Between the two sessions, the experimental mouse was removed from the arena, and was placed back into its home cage for approximately one min. The video-tracking data from both the “no target” and “target” conditions were used to determine the time spent by the experimental mouse in the “interaction zone” (a 5-cm-wide corridor surrounding the cage).

### Immunohistochemical staining

The Balb/c mice were deeply anaesthetized with 2% pentobarbital and perfused intracardially with phosphate-buffered saline (PBS; pH 7.4), followed by 4% paraformaldehyde in PBS. Their brains were then removed and fixed in the same fixative overnight at 4 °C, and then cryoprotected in 30% sucrose in PBS for 24 ~ 48 h at 4 °C . Serial coronal sections (30 μm) were cut on a cryostat (Leca, Wetzler, Germany). Free-floating sections were pre-treated with 3% hydrogen peroxide in PBS for 20 min at 22 °C , washed in PBS, and incubated in a blocking solution composed of 0.3% TritonX- 100 and 5% normal rabbit or goat serum for 30 min at 22 °C.The sections were subsequently incubated with the primary antibody against MBP (1:500; SantaCruz Biotechnology, CA, USA), or GST-π (1: 200; Boster, Wuhan, China), in the blocking solution overnight at 4 °C. The above antibodies were used to examine the myelin sheath (anti-MBP) and mature oligodendrocyte (anti-GST-π), respectively. Sections were then rinsed in PBS and incubated in biotinylated secondary antibodies (1:1000; VectorLabs, Burlingame, CA, USA) for 2 h at 22 °C. Following rinses in PBS, sections were incubated in avidin-biotin-horseradish peroxidase solution from the Vectastain (Elite) ABC kits (VectorLabs, Burlingame, CA,USA) for 30 min at 22 °C. Finally, sections were developed with DAB kit (VectorLabs, Burlingame, CA, USA) following the manufacturer’s instructions.

### Image analysis

Coronal sections of mPFC between bregma 1.98 mm and 1.54 mm containing prelimbic cortex (PrL) and infralimbic cortex (IL) regions were photographed under a Nickon Eclipse-80i microscope (Nikon Instruments Inc., Guangzhou, China). Three sections with an interval of 170 μm from each animal were selected for quantitative analysis. Measurements were focused on a selected rectangular area of 0.29 mm^2^ in mPFC, and this counting box was consistently positioned in each mouse. The Image-Pro Plus software (version4.1, Fryer, Huntley, IL, USA) was used for taking measurements. The parameters include the percentage of MBP-positive area over the examined area and the density of GST-π positive cells.

### Statistical analysis

Data are expressed as mean ± sem. Repeated measures two-way ANOVA with stress and time as two independent factors was used to analyze the behavioral data obtained from open-field test and MRS data. The Fisher’s LSD test was used for post-hoc comparisons. The two-tailed *Student’s t-*test was performed to compare sets of data obtained in social avoidance test and immunohistochemical staining experiments. Differences were considered statistically significant at *p* < 0.05.

## Additional Information

**How to cite this article**: Zhang, H. *et al*. The recovery trajectory of adolescent social defeat stress-induced behavioral, ^1^H-MRS metabolites and myelin changes in Balb/c mice. *Sci. Rep.* **6**, 27906; doi: 10.1038/srep27906 (2016).
